# The Cross-Talk Between Gut Microbiota and Lungs in Common Lung Diseases

**DOI:** 10.3389/fmicb.2020.00301

**Published:** 2020-02-25

**Authors:** Dapeng Zhang, Sha Li, Ning Wang, Hor-Yue Tan, Zhimin Zhang, Yibin Feng

**Affiliations:** ^1^First Affiliated Hospital of Guangzhou Medical University, Guangzhou, China; ^2^School of Chinese Medicine, LKS Faculty of Medicine, The University of Hong Kong, Hong Kong, China

**Keywords:** lung diseases, allergy, asthma, chronic obstructive pulmonary disease, cystic fibrosis, lung cancer, gut microbiota, gut–lung axis

## Abstract

Emerging findings indicate there is a vital cross-talk between gut microbiota and the lungs, which is known as gut–lung axis. The gut disturbances in lung diseases including allergy, asthma, chronic obstructive pulmonary disease, cystic fibrosis and lung cancer were observed by extensive studies. Investigating how gut microbiota impact other distant organs is of great interest in recent years. Although it has not been fully understood whether the disturbance is the cause or effect of lung diseases, alterations in the gut microbial species and metabolites have been linked to changes in immune responses and inflammation as well as the disease development in the lungs. In this article, we systemically review the role and mechanisms underlying the changes in the constituent of gut microbiota and metabolites in lung diseases. In particular, the roles of gut–lung axis in mediating immune responses and reshaping inflammation are highlighted. Furthermore, we discuss the potential of strategies to manipulate the gut microbiota and metabolites as the therapeutic approach for lung diseases.

## Introduction

**FIGURE 1:**
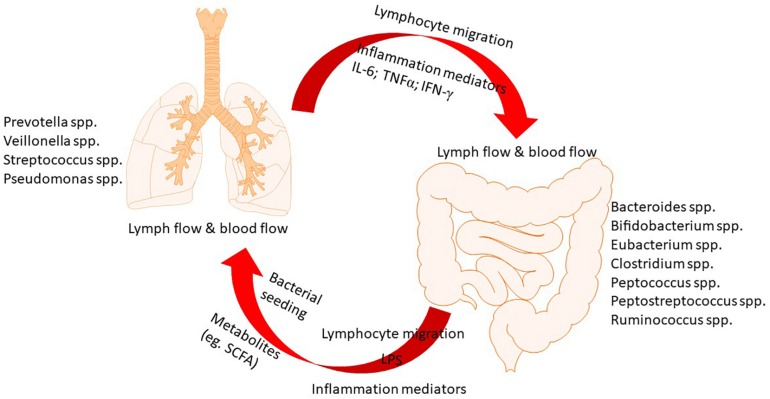
The cross-talk between gut and lung.

Microbes that inhabit in both gut and lung live in a mutualistic manner with the host. They benefit from a stable nutrient-rich microenvironment and also exert important functions such as fermentation of dietary components. Increasing evidence indicates the critical role of constitutive sensing of microbes and their metabolites to maintain the homeostasis of the immune system (Budden et al., 2017). In the microbial communities of gut, Bacteroidetes and Firmicutes are predominant while Bacteroidetes, Firmicutes, and Proteobacteria predominate in the lung. At the phylum level, the predominant microbial communities are similar in gut and lung (Marsland et al., 2015). However, in terms of species level, they are significantly different. As the largest and most diverse community of the mammalian microbiome, there are about 10^14^ bacteria colonize in the intestinal tract, which have been studied most extensively (Hillman et al., 2017). Emerging evidence has revealed that dysbiosis of gut microbiota is associated with various local and distant chronic diseases. A balanced microbial community in the gut is of great importance in immune function and health (McAleer and Kolls, 2018). The gut microbiota has been shown to affect pulmonary immunity through a vital cross-talk between gut microbiota and the lungs, which is referred to as gut–lung axis (Keely et al., 2012). This axis allows for the passage of endotoxins, microbial metabolites, cytokines, and hormones into the bloodstream connecting the gut niche with that one of the lung. Notably, the gut–lung axis is bidirectional, as shown in Figure 1. When the inflammation occurring in the lung, the lung-gut axis can induce changes in the blood and gut microbiota (Dumas et al., 2018).

An emerging area of intense interest is the role of gut–lung axis in the pathogenesis of lung diseases (Budden et al., 2017). Increasing studies indicated that alterations in the gut microbial species and metabolites have been linked to changes in immune responses and inflammation as well as the disease development in the lungs. For example, the risk of developing allergic airway disease increased due to antibiotic-caused changes in the gut microbiota in early life, facilitating our understanding of the links between exposure to microbiota and airway allergy (Noverr et al., 2005; Russell et al., 2012). The mechanisms by which the gut microbiota influences on the immune responses and inflammation in the lungs, and vice versa, are being extensively studied. The involvement of regulatory T cell subsets (Ohnmacht, 2016; Lee and Kim, 2017; Luu et al., 2017) and Toll-like receptors (TLRs) (O’Dwyer et al., 2016; Wang et al., 2018), inflammation cytokines and mediators (Scales et al., 2016), surfactant protein D (Du et al., 2019) and several other factors have been proposed as the underlying mechanisms, but many details unknown. Nevertheless, novel therapeutic strategies that target manipulation of gut microbiome by anti-biotics, probiotics, prebiotics, natural products, or diets have been attempted in various lung diseases by clinical and laboratory studies. In present article, we summarize the emerging role of gut–lung axis in a variety of common lung diseases, review the therapeutic strategies targeting manipulation of gut microbiome. We aim to update the information and evidence of this hot field, highlighting gaps in our knowledge and the potential for treatment of lung diseases via gut–lung axis.

## The Pathogenic Role of Gut Microbiota in Common Lung Diseases

### Asthma

Asthma is a common chronic respiratory diseases, which affects people of all ages but usually begins in childhood. It is a complex disease having multiple phenotypes with different pathophysiological and clinical characteristics (Gensollen et al., 2016; Bush, 2019). Since immunity has been recognized to play a vital role in the pathogenesis of asthma, such as the involvement of regulatory T cell subsets and TLRs, a link between gut microbes and allergy was hypothesized. This hypothesis was gradually verified via the finding of increased asthma risk due to antibiotic exposure in the first year of life (McKeever et al., 2002; Stiemsma and Turvey, 2017). The immune alterations driven by microbial indicate that microbial exposures would affect the risk of asthma (Johnson and Ownby, 2017). In a study, it was found that 1-year-old children born to asthmatic mothers with an immature microbial composition had higher risk of asthma at age 5 years. This finding suggested that deficiency of microbial stimulation at the beginning of life might induce the inherited asthma risk, and sufficient maturation of the gut microbiome in this period may be beneficial to prevent (Stokholm et al., 2018). Low total diversity of the gut microbiota of infant during the first month of life was associated with asthma development in children at 7 years old (Abrahamsson et al., 2014). The diversity of the gut microbial in early life may avoid airway inflammation in asthma via mediating the balance of Th1/Th2 (Qian et al., 2017).

Then emerging evidence shows that maturation patterns of the gut microbiome affect the asthma risk for children. In a study, bacterial DNA isolated from the stool samples from 92 children diagnosed with asthma and 88 healthy children were detected. *Akkermansia muciniphila* and *Faecalibacterium prausnitzii* were found to be decreased in asthma group compared to healthy group. Both bacterial species may suppress inflammation via modulating secreted metabolites, such as increased IL-10 and decreased IL-12 (Demirci et al., 2019). The levels of inflammatory factors including C-reactive protein (CRP), tumor necrosis factor-alpha (TNF-α), and interleukin-6 (IL-6) in peripheral serum of children with asthma were significantly higher than those in the controls. In particular, CRP was correlated positively with the total load of intestinal bacteria and gastrointestinal symptom rating scale (GSRS) scores, indicating that with the increased levels of inflammatory factors in peripheral serum, the likelihood of gut dysbiosis and gastrointestinal incommensurate symptoms will rises in children with asthma (Zhang Y. et al., 2018). In addition to the altered constituent of gut microbiota, the change of related metabolites has also been noted. A significant reduction of the total content of the fatty acids and the absolute concentrations of the specific acids including acetate, butyrate and propionate, as well as the content of isoacids in the feces from patients with bronchial was observed compared to normal controls (Ivashkin et al., 2019). The gut microbiota patterns in adults with diagnosed asthma have also been studied. In a pilot study, significant relationships between gut microbiota composition, aeroallergen sensitization and lung function in asthmatic and non-asthmatic adults were observed (Begley et al., 2018).

**FIGURE 2:**
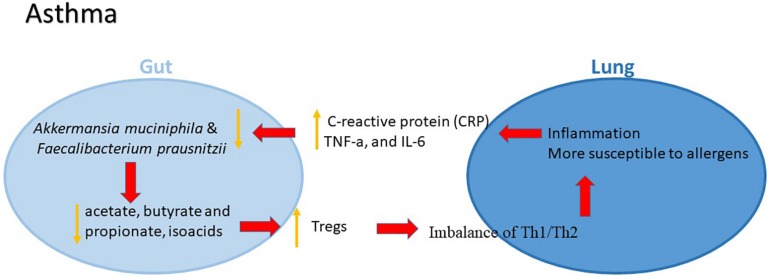
The potential role of gut–lung axis in asthma.

The mechanism by which gut microbiota influence the initiation and development of asthma, however, remains largely unknown. The microbes, bile salts and other immune stimuli from the digestive tract might play a vital role in mucosal immunity of the respiratory system (Belkaid and Hand, 2014). The epithelial mucosa and dendritic cells as well as antimicrobial peptides secreted by immune cells are major effectors in the response to environmental agents in the airway lumen (Elenius et al., 2017). The epithelium controls the local respiratory immune activities that are also mediated by thymic stromal lymphopoietin, IL-25 and IL-33, which might lead to a Th2 type inflammation, thus facilitating the development of asthma (Gauvreau et al., 2014). The gut microbiome contributes to the generation of Tregs, making the lung more susceptible to oral allergens (Penders et al., 2007). Tregs generated in the periphery, known as induced Tregs (iTreg), are predominantly stimulated in the mesenteric lamina propria Peyer’s patches, and lymph nodes of the small and large intestines (Josefowicz et al., 2012). Increasing evidence suggested that gut microbiome has an important role in coordinating both the innate and adaptive immunity that is involved in the development of asthma, whereas the underlying molecular mechanisms are needed to be further identified (Figure 2).

### Chronic Obstructive Pulmonary Disease (COPD)

Chronic obstructive pulmonary disease (COPD) is a prevalent chronic inflammatory lung disease that causes obstructed airflow resulting from chronic aeropollutant exposure, primarily from smoking (Rabe and Watz, 2017). Increasing evidence indicated that gut-liver-lung axis plays a vital role in the pathogenesis of COPD (Young et al., 2016). The liver is a key organ to orchestrate innate immunity and participates actively in innate immune responses in other sites including gut and lung through generation of inflammatory cytokines and mediators (Inatsu et al., 2009; Jenne and Kubes, 2013). The elevated systemic inflammatory mediators due to overactive innate immune responsiveness, such as C-reactive protein (CRP) and IL-6, directly contributes to both morbidity and mortality in COPD. IL-6 activates the innate immune response to maintain the systematic inflammation as part of the normal immune in response to smoking or bacterial invasion exposure. In response to elevated serum IL-6, acute-phase proteins such as CRP is generated within the liver (Inatsu et al., 2009). Liver might amplify innate immune responsiveness in respiratory system when exposes to bacteria or smoking through facilitating alveolar macrophage release of IL-6 and acute-phase proteins.

**FIGURE 3:**
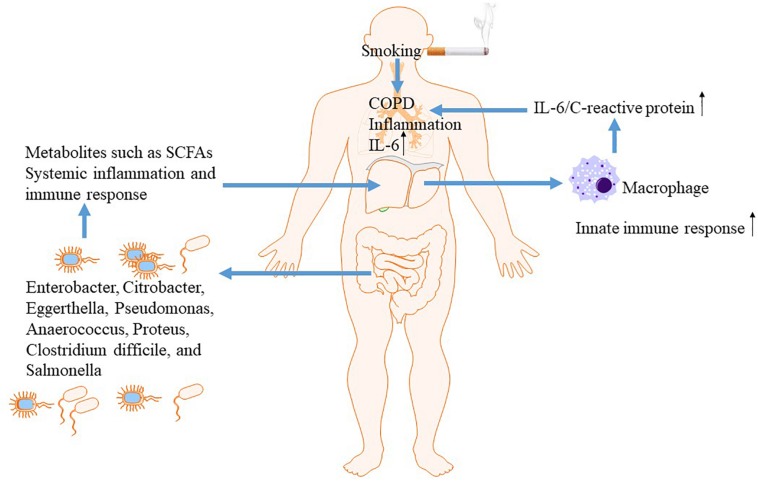
The gut-liver-lung axis in COPD.

Then, growing evidence suggested that gut is linked to the lungs and liver via dietary factors (Kuo, 2013). Epidemiological studies found that a diet containing rich fiber was associated with decreased risk of COPD and better lung function (Kan et al., 2008; Varraso et al., 2010). Fiber is the remaining substance in food that cannot be digested by the body, acting as a source of nutrition for most of the beneficial bacteria in the intestine. Its major physiology function is enhancing the activity of beneficial bacteria in the gut and maintaining intestinal health (Kranich et al., 2011). Thus, gut and resident microbiota may play an essential role in the pathogenesis underlying COPD. The mechanism underlying the protective effect of high-fiber diets on COPD might be associated with modulation of innate immunity and systemic inflammation, epithelial integrity from microbial invasion, and stimulating beneficial bacteria to generate small chain fatty acids (SCFAs) (Kan et al., 2008; Varraso et al., 2010; Park et al., 2011; Chuang et al., 2012; Macia et al., 2012). Among subjects with smoking exposure, lung function was improved via increased intake of dietary fiber, further supporting the vital role of gut-liver-lung axis in COPD (Figure 3).

The change of gut microbiome and function of intestinal mucosal barrier was observed in COPD by experimental and clinical studies. In contrast to control subjects, the gut microbiota in COPD patients is characterized by the presence of representatives of the Pro-teobacteria, such as *Enterobacter cloacae*, *Citrobacter*, *Eggerthella*, *Pseudomonas*, *Anaerococcus*, *Proteus*, *Clostridium difficile*, and *Salmonella* (Charlson et al., 2011). In a COPD rat model exposure to cigarette smoke for 6 months, structural and dysfunctional changes in the intestinal mucosal barrier were observed, associating with the aggravated intestinal inflammatory responses (Xin et al., 2016). Additionally, COPD has a high associated risk for cardiovascular disease and death due to an infectious cause. The gut, microflora-dependent metabolite trimethylamine-*N*-oxide (TMAO), as a dietary-associated risk factor for incident cardiovascular events (Troseid et al., 2015), has been demonstrated to be predictive for adverse clinical outcomes in patients with COPD regardless type of exacerbation. Significantly higher median admission TMAO levels was observed in patients with shorter lifetime compared with survivors. Increased levels of circulating TMAO were associated with long-term all-cause mortality in exacerbated COPD patients. Nutritional interventions targeting reduction of TMAO levels might be strategies for the management of COPD and related cardiovascular disease (Ottiger et al., 2018).

### Respiratory Infections

The pulmonary microbial community and gut microbiota have been observed to be considerably changed in a variety of respiratory disorders caused by a wide range of airborne pathogens (Dumas et al., 2018). It is believed, these commensal bacteria affect the process of the airway infectious diseases via local or distal immune modulation. The use of probiotics, which has beneficial effects on the resistance of pathogens and activation of host immunity, shows promising effect in respiratory disorders (Alexandre et al., 2014). Although whether microbial dysbiosis is a cause or a consequence of respiratory infectious disease remains unclear, gut microbiota, as the most diverse community of the mammalian microbiome, has been demonstrated to show a vital impact on host immune response in both sites (Pickard et al., 2017; Mendez et al., 2019). The role of gut–lung axis in airway infectious diseases such as caused by tuberculosis bacillus, virus, fungi and other bacteria was focused in present review.

#### Tuberculosis

The weakening in immune health status is related with tuberculosis infection (Tarashi et al., 2018). Gut microbiome, which is implicated in the modulation of host immunity and metabolism, has been intensively studied in patients with tuberculosis (Luo et al., 2017; Maji et al., 2018; Hu et al., 2019a). In patients with active tuberculosis, significantly enriched butyrate and propionate-producing bacteria like *Roseburia*, *Faecalibacterium*, *Phascolarctobacterium*, and *Eubacterium* were observed compared to those healthy household controls. In a recent study, it was found that the gut microbiota of patients with active tuberculosis were mostly featured by the striking decrease of short-chain fatty acids (SCFAs)-producing bacteria as well as associated metabolic pathways (Saitou et al., 2018). This discrepancy might result from the difference of ethnics, body mass index, disease status and some other parameters of selected patients from different studies. Further study with large sample size is needed. A classification model based on the abundance of three species, *Haemophilus parainfluenzae, Roseburia inulinivorans*, and *Roseburia hominis*, performed well for discriminating between healthy and diseased patients, as assessed by the receiving operational curve (ROC) analysis, achieved the area under the ROC (AUC) of 84.6%, and a 95% confidence interval (CI) of 0.651–0.956. Additionally, the healthy and diseased states can be distinguished by SNPs in the species of *B. vulgatus*. The decreased biosynthesis of amino acids and vitamins in favor of augmented metabolism of butyrate and propionate was found in active tuberculosis patients by functional analysis. In addition to altered gut microbiota, intestinal lesions in many active tuberculosis patients were also detected by using small bowel capsule endoscopy (Saitou et al., 2018). The differences of gut microbiota in new tuberculosis patients, and recurrent tuberculosis patients were also characterized. Proteobacteria and *Actinobacteria* were significantly enriched whereas Bacteroidetes, having various beneficial commensal bacteria, was reduced in the gut of patients with recurrent tuberculosis compared to healthy controls. The genus *Prevotella* and *Lachnospira* were significantly decreased in both the new and recurrent tuberculosis patient groups compared with the healthy subjects (Luo et al., 2017). The variation of gut microbiota during the course of treatment was also investigated in subjects with tuberculosis. A rapid and significant change in the community structure was observed after anti-tuberculosis treatment. The relative abundance of members of phylum Firmicutes and genus *Clostridiales* reduced significantly, whereas genus *Bacteroides*, including *Bacteroides fragilis* and *Bacteroides OTU230*, was increased. OTU2972 and OTU8 from family Erysipelotrichaceae of the phylum Firmicutes showed a significant increase in 1 week after the beginning of treatment, while the other members of this family reduced (Hu et al., 2019b). After treatment of anti-tuberculosis drugs for a month, significant alteration in metagenome gene pool was observed, suggesting the recovery in functional ability (Maji et al., 2018). In another study, it was found that the commensal bacterium Helicobacter pylori may protect lung from tuberculosis disease, while H. hepaticus may increase susceptibility to *Mycobacterium tuberculosis* infection (Tarashi et al., 2018). Thus, these findings from pilot studies indicate that microbial dysbiosis may contribute to pathophysiology of lung tuberculosis infection and the beneficial role of the intestinal microbiota against lung infections throws light on promising therapeutic and immunization strategies.

In a recent study, the mechanism underlying the interaction of intestinal microbiota and lung in immune response to *Mycobacterium tuberculosis* infection has been explored (Negi et al., 2019). It was found that gut dysbiosis induced by antibiotics reduced the lung macrophage inducible C-type lectin (mincle) expression with a concomitant increase in *Mycobacterium tuberculosis* survival. Furthermore, antibiotics elevated the population of regulatory T cells (Tregs) while reduced the effector and memory T cell population in the lungs. Mice receiving antibiotics showed low mincle expression on lung dendritic cells. These impaired dendritic cells caused decreased ability to activate naïve CD4 T cells, resulting in *Mycobacterium tuberculosis* survival. Administration of trehalose-6,6-dibehenate, a mincle ligand, could enhance the function of lung dendritic cells and T cell response. Therefore, gut microbial components, serving as ligand for a variety of pattern recognition receptors on immune cells, orchestrate host immunity involved in *Mycobacterium tuberculosis* infection (Negi et al., 2019).

#### Viral and Fungal Infections

The effects of alteration in intestinal microbiota on respiratory viral infections have been studied in recent years (Berger and Mainou, 2018; Hanada et al., 2018; Li et al., 2019). In a study, C57BL6 mice were treated with streptomycin before or during infection with murine paramyxoviral virus type 1, Sendai virus (SeV) (Grayson et al., 2018). A significant reduction in intestinal microbial diversity without impact on lung microbiota was observed after the administration of streptomycin. Reduction in diversity in the gastrointestinal tract was followed by greatly increased mortality to respiratory viral infection. This increase in mortality to respiratory viral infection was associated with a defected immune response characterized by increased lung IFN-γ, IL-6 and CCL2, and decreased count of Tregs in lung and intestinal. Neutralization of IFN- γor adoptive transfer of Tregs significantly reduced increased mortality (Grayson et al., 2018). In another study using murine models of influenza virus and respiratory syncytial virus (Rangelova et al., 2019), significantly altered gut microbiota diversity, with a decrease in Firmicutes phyla abundance and an increase in Bacteroidetes was observed in mice infected with influenza virus or RSV. Viral lung infections also caused an increase in colonic Muc5ac levels and fecal lipocalin-2, indicating low-grade inflammation in gut (Groves et al., 2018). In patients undergoing allogeneic hematopoietic stem cell transplantation (allo-HCT), respiratory viral infections are occurring frequently, which could further progress to lower respiratory tract infection. Higher population of butyrate-producing bacteria in intestinal microbiota contributes to the risk of lower respiratory tract infection following viral infection in patients with allo-HCT (Haak et al., 2018).

The diversity of the gut microbiota was significantly changed by lung infection with *Pneumocystis murina* (*P. murina*). In mice without CD4 + T cell infected with *P. murina* showed gut microbial community that was significantly distinct from normal mice with *P. murina* infection, indicating that lacking of CD4 + T cells may alter the gut microbiota as well in *Pneumocystis* pneumonia. Furthermore, the functional potential for carbohydrate, energy, and xenobiotic metabolism, as well as signal transduction pathways of intestinal microbiota was altered in the setting of *Pneumocystis* pneumonia (Samuelson et al., 2016). In the setting of pulmonary fungal infection, it was found that anti-TNFα treatment promoted the migration of dendritic cells from the gut to the lung that enhanced Tregs, facilitating susceptibility to pulmonary *Histoplasma capsulatum* infection (Tweedle and Deepe, 2018). Together, gut microbiota regulate immune responses at distant mucosal sites and is able to affect mortality in response to viral lung and fungal infection (Grayson et al., 2018).

#### *Staphylococcus aureus* Pneumonia

Streptococcus pneumoniae is a common pathogen responsible for great morbidity and mortality worldwide (Henriques-Normark and Tuomanen, 2013). The protective role of gut microbiota during pneumococcal pneumonia has been revealed several years ago (Schuijt et al., 2016). It could improve primary alveolar macrophage function. In particular, it was found that segmented filamentous bacteria (SFB) contributes to resistance against *Staphylococcus aureus* (*S. aureus*) in immunocompromised host. In Rag(−/−) C57BL/6 mice with adaptive immune deficiency, SFB affected lung protection by mediating innate immunity. A significant decrease in lung neutrophils in the resolution phase of *S. aureus* infection, along with decreased expression of CD47 that impedes phagocytosis of apoptotic cells, was demonstrated in SFB-colonized Rag(−/−) mice. SFB stimulated inflammatory neutrophils to shift toward pro-resolution neutrophils via down-regulating CD47. Thus, in immunocompromised hosts, gut commensal SFB may offer much-needed defense partly through promoting neutrophil resolution in Pneumococcal pneumonia (Felix et al., 2018). In normal C57BL/6 mice, SFB stimulates the induction of pulmonary type 17 immunity and resistance to *S. aureus* pneumonia (Gauguet et al., 2015).

The mechanism by which gut microbiota protect lung from *S. aureus* infection has been explored recently. Gut microbiota disruption caused by antibiotics decreases pulmonary resistance to *S. aureus* resulting from impaired Toll-like receptor 4 (TLR4) function (Wang et al., 2018). Mast cells are involved in regulation of lung-gut axis during *S. aureus* pneumonia. After *S. aureus* infection, decreased lung inflammation, reduced level of cathelicidin-related antimicrobial peptide (CRAMP), higher bacterial lung load and intestinal flora dysbiosis were found in mast cells-deficient mice compared with wild-type mice. Adoptive transfer of mast cells from bone marrow into the lung successfully rebuilt the host defense against *S. aureus* and led to recovery of intestinal dysfunction induced by *S. aureus* pneumonia. Additionally, treatment of exogenous CRAMP also significantly improved the host immunity to against bacterial in the lungs of mice without mast cells (Liu C. et al., 2019).

In the setting of *S. aureus* pneumonia-induced sepsis, the mechanism by which intestinal injury is caused has been intensively studied. A microRNA that has been identified in various cancers, miR-182-5p, contributes to intestinal injury via targeting surfactant protein D (SP-D). Moreover, SP-D was reduced significantly in pneumonia mice models. It binds with the bacterial pathogens and eradicate the pathogens and apoptotic bodies, playing vital role in the regulation of immune responses (Du et al., 2019). In another study, it was demonstrated that SP-A and SP-D diminished severity of *S. aureus* pneumonia and gut mucosal injury. The potential mechanism underlying their protection are attributed to enhanced pulmonary clearance of *S. aureus*, decreased expressions of caspase-3 and Bax/Bcl-2 as well as reduced activation of the NF-κB signaling pathway in gut during pneumonia (Du et al., 2016).

### Cystic Fibrosis

**FIGURE 4:**
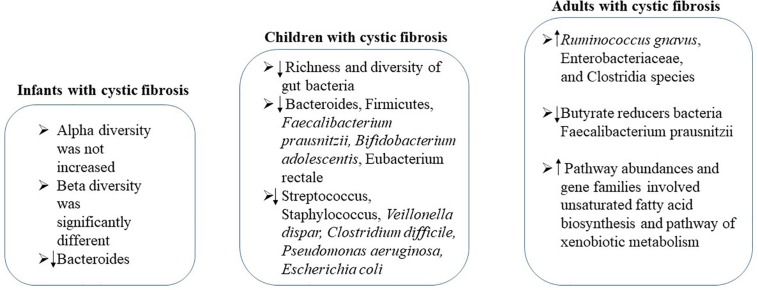
The changes of gstrointestinal microbiota in cystic fibrosis patients at different ages.

Cystic fibrosis is a genetic disease that affects mostly the lungs and digestive system (Haack et al., 2013). Increasing evidence shows a link between gastrointestinal microbiota and progression of lung disease in cystic fibrosis (Schippa et al., 2013; Li and Somerset, 2014; Nielsen et al., 2016; Rogers et al., 2016; Burke et al., 2017; Fouhy et al., 2017) (Figure 4). In infants with cystic fibrosis, alpha diversity was not increased over the first year of life as expected in control cohort, while the beta diversity of these two cohorts was significantly different, associating with airway exacerbations (Antosca et al., 2019). Compared with controls, levels of *Bacteroides*, a bacterial genus related with immune modulation, were decreased over the first year of life. *Bacteroides* species supernatants could reduce the generation of IL-8 from intestinal cell lines, indicating the alteration in the gut microbiota influences the inflammation in cystic fibrosis (Antosca et al., 2019). During this critical window of immune programing, interventions targeting to establish a healthy state of gastrointestinal microbiota in infants with cystic fibrosis may be beneficial for lifelong health (Antosca et al., 2019).

A significant decrease in the richness and diversity of gut bacteria of cystic fibrosis children in age from 0.87 to 17 years was detected. Strategy to correct the reduction of bacterial diversity and maintain bacterial physiological function should be performed no later than early childhood (Nielsen et al., 2016). In children with cystic fibrosis, reduced abundances of *Bacteroides*, Firmicutes, *Faecalibacterium prausnitzii, Bifidobacterium adolescentis* and *Eubacterium rectale*, along with increased *Streptococcus*, *Staphylococcus*, *Veillonella dispar, Clostridium difficile, Pseudomonas aeruginosa*, and *Escherichia coli* were observed, showing similarities as Crohn’s disease (de Freitas et al., 2018; Enaud et al., 2019). In particular, the increase of Streptococcus in children with intestinal inflammation seems to be specific to cystic fibrosis, suggesting the involvement of gut–lung axis in its pathogenesis (Enaud et al., 2019). Furthermore, the intestinal inflammation in patients with cystic fibrosis might be related to alterations of the composition of the gut microbiota (de Freitas et al., 2018). It has been demonstrated that the gut microbiota pattern is caused by cystic fibrosis transmembrane conductance regulator (CFTR) function impairment (De Lisle, 2016; Vernocchi et al., 2018). The interaction of cystic fibrosis host–gut microbiota provides new insights to the treatments or interventions to enhance intestinal function and nutritional status of children suffering from cystic fibrosis.

In stable adults with cystic fibrosis, the altered intestinal microbiota functionality has also been demonstrated (Duytschaever et al., 2013; Huang and LiPuma, 2016; Fouhy et al., 2017; Vernocchi et al., 2017). Significantly decreased Bacteroidetes and increased Firmicutes were observed in cystic fibrosis adults (Burke et al., 2017; Fouhy et al., 2017). By fecal proteomics study, it was demonstrated that the abundances of *Ruminococcus gnavus*, Enterobacteriaceae, and *Clostridia* species was increased while butyrate reducers *Faecalibacterium prausnitzii* was decreased in patients with cystic fibrosis (Debyser et al., 2016). Pathway abundances and gene families involved in unsaturated fatty acid biosynthesis and pathway of xenobiotic metabolism were higher in cystic fibrosis adults compared to that of healthy controls (Fouhy et al., 2017). Furthermore, remarkable differences in the metabolites from gut microbiota also occurred between cystic fibrosis patient and healthy controls (Fouhy et al., 2017).

### Lung Cancer

Lung cancer is one of the deadliest malignancies with the fastest growing morbidity and mortality worldwide. The role of gut microbiome in various cancers have been reported by numerous studies (Feng et al., 2018). In patients with cancer, the composition of the gut microbiota often develops significantly different from that of normal controls. In the setting of lung cancer, previous studies mainly focus on the impact of lung microbes, which have direct contact with the lung tissues. Until recently, the relationship between gut microbiome and lung cancer has been explored. It was found that the increased levels of *Enterococcus* sp. and decreased levels of *Bifidobacterium* sp. and *Actinobacteria* sp. are associated with lung cancer. Furthermore, the impairment of the normal function of the gut microbiome impacts on the progression of lung cancer (Zhuang et al., 2019). In another study, lower levels of *Dialister, Enterobacter, Escherichia–Shigella, Fecalibacterium*, and *Kluyvera* but higher levels of *Veillonella*, *Bacteroides*, and Fusobacterium compared to that of the controls were found (Zhang W. Q. et al., 2018). The correlations between the relative abundance of the above-mentioned bacterial genera and systemic inflammation-related markers including platelet-to-lymphocyte ratio (PLR), neutrophil-to-lymphocyte ratio (NLR), prognostic nutritional index (Fontalis et al., 2019), and lymphocyte-to-monocyte ratio (LMR) were analyzed. The results showed that *Enterobacter* and *Escherichia–Shigella*, were correlated positively with serum NLR level, while *Dialister* was negatively correlated with contents of PLR and NLR in the serum. Moreover, *Dialister* was also correlated with serum levels of CTLA-4 and IL-12 (Zhang W. Q. et al., 2018).

Gut microbiota variation is correlated with the risk of immune-related diarrhea in lung cancer patients after the treatment of anti-programed cell death protein-1 (anti-PD-1) antibodies. *Phascolarctobacterium*, as well as *Bacteroides* and *Parabacteroides* were more abundant whereas *Veillonella* was lower in patients without diarrhea (Liu T. et al., 2019). Given the vital role of gut microbiota in shaping immune responses, the influence of antibiotics on the efficacy of immunotherapy in patients with lung cancer was studied by a retrospective analysis. Although there is a certain trend showing the negative influence of antibiotic use, no statistical significance was observed between survival and prior antibiotic use in non-small cell lung cancer patients treated with nivolumab (Hakozaki et al., 2019).

## Manipulation of Gut Microbiota in the Treatment of Common Lung Diseases

Given the role of gut microbiota, whether manipulation of gut microbiota represents a promising therapeutic strategy for lung diseases has been validated by increasing clinical and experimental studies. Interventions including anti-biotics, probiotics, prebiotics, and natural products or diets that target manipulation of gut microbiota were attempted in subjects with lung diseases (Table 1). Major attempts have been made in patients with cystic fibrosis or lung cancers. In this section, we discussed and reviewed herbal plants targeting manipulation of gut microbiota via regulating inflammation and immune-response, acting as probiotics and prebiotics, supplementing micronutrients and exerting cytotoxic effects on cancer cells in the treatment of common lung disease.

**TABLE 1:** Interventions targeting manipulation of gut microbiota in lung diseases.

Intervention	Study type	Effects and outcomes	Underlying mechanisms	References
Multispecies probiotic	Randomized, placebo-controlled, double-blind study COPD patients	Showed a modest effect on the bacterial subgroups but no effect on occurrence of diarrhea-like bowel movements and the composition of the dominant fecal microbiota	Resulted from an existing imbalance of the microbiota	Koning et al., 2010
*Lactobacillus rhamnosus* GG	Mice model with *Pseudomonas aeruginosa* pneumonia	Reduced inflammation and maintained intestinal barrier homeostasis	Enhancing gut mucin expression/barrier formation, reducing apoptosis, and improving cell proliferation	Khailova et al., 2017
Flavonoid	Clinical study, adult patients with cystic fibrosis	Associated with gut microbita variations, such as correlating with the genus Actinomyces and family Actinomycetaceae (Actinobacteria)	With potential consequences for metabolism, immune function, and inflammation	Li and Somerset, 2018
Vitamin D	Clinical and laboratory study, cystic fibrosis	Developed a healthy gut microbiota, maintained the integrity of the gut mucosal barrier, allowed beneficial bacteria to outcompete opportunistic pathogens	Enhancing intercellular junctions, reducing pro-inflammatory cytokines such as IL-8, inhibiting apoptosis of intestinal epithelial cells	Kanhere et al., 2018
Micronutrient such as vitamin C, vitamin E, niacin, beta-carotene, and riboflavin	Clinical study, adults with cystic fibrosis	Correlated negatively with *Bacteroides*; positively correlated with Firmicutes	Not yet clarified	Li et al., 2017
Streptomycin	A cystic fibrosis mouse model	Altered the intestinal microbiome, pulmonary T cell profile and airway hyperresponsiveness	By reducing the intestinal bacterial overgrowth in Cftr(tm1UNC) mice and by affecting *Lactobacillus* levels	Bazett et al., 2016
A *Lactobacillus reuteri* probiotic preparation	A double blind prospective study	Improvement in the gastrointestinal health and decrease of the calprotectin levels	Reduction in gamma-proteobacterial populations	del Campo et al., 2014
Diet therapies	Clinical study, patients with cystic fibrosis	Improving symptom associated with chronic systemic inflammation		Li and Somerset, 2014
*Lactobacillus* GG	A Randomized Clinical Trial, children with cystic fibrosis	Reduced microbial richness and intestinal inflammation	Partially restored intestinal microbiota	Bruzzese et al., 2014
Probiotics	A Randomized Controlled Trial, patients with cystic fibrosis	Reduced number of pulmonary exacerbations and improving quality of life	Immunomodulatory and anti-inflammatory	Jafari et al., 2013
Pentaherbs formula	Allergic Asthma, ovalbumin-induced allergic asthma mice model	Reduce airway hyperresponsiveness, airway wall remodeling and goblet cells hyperplasia, suppressed pulmonary eosinophilia and asthma-related cytokines IL-4 and IL-33, altered the microbial community structure and the short chain fatty acids content in the gut of the asthmatic mice.	Suppressing various immune effector cells	Tsang et al., 2018
*Bifidobacterium longum* BB536	Randomized, double-blind, parallel and placebo-controlled study	Potential protective effects	Higher abundance of the genus *Faecalibacterium* associating with anti-inflammatory and immuno-modulatory properties	Lau et al., 2018
Recuperating Lung Decoction	Rats with Allergic Bronchial Asthma	Increased *Lactobacillus* and *Bifidobacterium* spp.		Kong et al., 2016
Diet enriched with acidic oligosaccharides	Mice with pulmonary *Pseudomonas aeruginosa* infection	Led to increased bacterial clearance after P. aeruginosa infections, limited the number and severity of pulmonary exacerbations	Stimulating the growth of species involved in immunity development, such as *Sutturella wadsworthia*, and *Bifidobacterium* species, increased the generation of butyrate and propionate.	Bernard et al., 2015
*Enterococcus faecium κ*-50 and *Saccharomyces cerevisiae* 14*κ*	Lewis lung carcinoma, mice	Inhibited metastasis		Tanasienko et al., 2005
*Bifidobacterium infantis*-mediated sFlt-1 gene transferring system	Lung cancer, C57BL/6 mice	Inhibition of the tumor growth and prolonged survival time		Zhu et al., 2011
*Bifidobacterium infantis*-mediated soluble kinase insert domain receptor	Lung cancer, C57BL/6 mice	Tumor growth Inhibition and prolonged survival time	Increasing the necrosis rate of the tumor	Li et al., 2012
Commensal microbiota	Lewis lung carcinoma and B16F10 lung metastases, C57BL/6 mice	Microbiota modifications	γδT17 immune cell-dependent mechanism	Cheng et al., 2014
*L. acidophilus*	Lung cancer, C57BL/6 mice	Increased anti-tumor effect of cisplatin and survival rates		Gui et al., 2015
*Lactococcus lactis* NK34	Lung cancer cells, *in vitro*	Cytotoxic effect		Mortaz et al., 2013
*Lactococcus lactis* KC24	Lung cancer cells, *in vitro*	Strong cytotoxic effect		Sharma et al., 2018
*Bacteroides fragilis*	Non-small cell lung carcinoma, mice	Anti-tumor effect	Immunostimulatory effects of CTLA-4 blockade	Vetizou et al., 2015
*Fermented milk by L. casei CRL 431*	Lung metastasis, BALB/c mice	Reduced tumor growth and lung metastasis	Decreasing macrophages infiltration and increasing CD4 + CD8 + lymphocytes	Aragon et al., 2015
Kefir (a probiotic-containing fermented milk product)	Lung metastasis, BALB/c mice	A reduction in metastasis to lung	increasing T helper cells and cytotoxic T cells	Zitvogel et al., 2017
*Enterococcus hirae* and *Barnesiella intestinihominis*	Advanced lung, mice	Increased cyclophosphamide-anticancer effects	Promoting the infiltration of IFN-γ-producing γδT cells in cancer lesions	Dasari et al., 2017

### Regulating Inflammation and Immune-Response

Herbal plants, which show regulation effect on gut microbiota, have also been used in asthma. Pentaherbs formula, comprising five traditional Chinese herbal medicines *Moutan Cortex*, *Phellodendri Cortex*, *Menthae Herba*, *Atractylodis Rhizoma*, and *Lonicerae Flos*, shows anti-inflammatory and anti-allergic potential via suppressing a variety of immune effector cells. In ovalbumin-induced allergic asthma mice model, it decreased airway hyperresponsiveness, airway wall remodeling and goblet cells hyperplasia, inhibited pulmonary eosinophilia and asthma-related cytokines IL-4 and IL-33. Notably, it altered the gut microbial community structure and metabolites such as short chain fatty acids level of asthmatic mice. Herbal medicines can alleviate symptoms of allergic asthma via impacting on gut microbiota and related metabolites (Tsang et al., 2018).

In a randomized double-blind prospective trial, the supplementation of probiotic reduced upper respiratory infection rate by stimulating immune response (Zhang H. et al., 2018). A probiotic *Bifidobacterium longum* 5(1A) was demonstrated to protect mice against lung infection caused by Klebsiella pneumonia. It induced faster resolution of inflammation by increased production of IL-10, decreased lung injury with significant decrease of bacterial burden. The underlying mechanism is partly attributed to activation of the TLRs adapter protein Mal (Vieira et al., 2016). In a randomized clinical trial, a galacto-oligosaccharide/polidextrose enriched formula protected infants who were born to atopic parents from respiratory infections. It increased the colonization of the protective bacteria including *Bifidobacteria* and *Clostridium* cluster I (Ranucci et al., 2018).

### Acting as Probiotics and Prebiotics

Probiotics, as immunomodulatory and anti-inflammatory substances, are useful nutritional supplements on improving quality of life and decreasing number of pulmonary exacerbations in patients with cystic fibrosis (Jafari et al., 2013). *Bifidobacterium longum* BB536, a multifunctional probiotic, has been shown to relieve upper respiratory diseases with intestinal microbiota modulating properties by a randomized double-blind study of Malaysian pre-school children aged 2–6 years old (Lau et al., 2018). The abundance of the genus *Faecalibacterium* associating with anti-inflammation and immuno-modulation was significantly increased by the BB536 treatment compared to the placebo group. As concluded by a systematic review, some randomized clinical trials suggested that the probiotic supplementation is beneficial for management of pulmonary exacerbation and intestinal inflammation in cystic fibrosis patients, however the evidence is limited and high quality of study is needed (Nikniaz et al., 2017).

In a randomized, placebo-controlled, double-blind study, a multispecies probiotic or placebo was treated to COPD patients who received antibiotics for respiratory tract infection (Koning et al., 2010). The multispecies probiotic showed a modest effect on the bacterial subgroups whereas it affected neither the occurrence of diarrhea-like bowel movements nor the composition of the dominant fecal microbiota (Koning et al., 2010). The non-effective of probiotic might resulted from an existing imbalance of the microbiota (Koning et al., 2010). Another herbal formula, recuperating lung decoction, increase the level of intestinal lactic acid-producing bacteria *Lactobacillus* and *Bifidobacterium* spp. in asthma model rats (Kong et al., 2016). *Lactobacillus* play a vital role in cystic fibrosis as a beneficial flora. In a Randomized Clinical Trial of children with cystic fibrosis, *Lactobacillus rhamnosus* GG partially restored intestinal microbiota, leading to reduced microbial richness and intestinal inflammation (Bruzzese et al., 2014). The administration of *Lactobacillus rhamnosus* GG via oral improved gut permeability and modulated inflammatory response and homeostasis of spleen and colon in FVB/N mice with *Pseudomonas aeruginosa* pneumonia. The mechanism underlying its protection is enhancing gut mucin expression, improving cell proliferation and reducing the pro-inflammatory cytokine expression (Khailova et al., 2017). A *Lactobacillus reuteri* probiotic preparation was demonstrated to improve gastrointestinal health and decrease the calprotectin levels due to the reduction of gamma-proteobacterial populations by a double blind prospective study of cystic fibrosis patients (del Campo et al., 2014). In a cystic fibrosis mouse model, antibiotic streptomycin treatment affected the intestinal microbiome, pulmonary lymphocyte profile and airway hyperresponsiveness. It reduced the overgrowth of intestinal bacterial in Cftr mice and principally altered *Lactobacillus* levels (Bazett et al., 2016).

Probiotics on elevating efficacy of anti-tumor medicines has been demonstrated. For example, *L. acidophilus* increased anti-tumor effect of cisplatin and increased survival rates of C57BL/6 mice with lung cancer (Gui et al., 2015). In another study, *Enterococcus hirae* and *Barnesiella intestinihominis* increased cyclophosphamide-anticancer effects in mice with advanced lung cancer through promoting the infiltration of IFN-γ-producing γδT cells in cancer lesions (Dasari et al., 2017).

Several probiotics exert effect on suppressing metastasis to lung via immune modulation. A probiotic-containing fermented milk product showed significant cytotoxic on 4T1 breast tumor cells and reduced metastasis to lung by increasing T helper cells and cytotoxic T cells (Zitvogel et al., 2017). In another research, the treatment of fermented milk by *L. casei* CRL 431 on BALB/c mice with breast cancer led to the suppression of tumor growth with decreased extravasation of tumor cells and tumor vascularity, as well as lung metastasis. The mechanism was attributed to altered immune response such as increasing CD4 + and CD8 + lymphocytes and decreasing infiltrated macrophages (Aragon et al., 2015). *Enterococcus faecium κ*50 and *Saccharomyces cerevisiae* 14*κ* inhibited metastasis in mice with Lewis lung carcinoma (Tanasienko et al., 2005). In melanoma lung metastasis, commensal microbiota transplantation reduced the number of metastatic lung foci through a γδT17 immune cell-dependent mechanism (Cheng et al., 2014).

Furthermore, *Bifidobacterium infantis*, a recombinant probiotic bacteria, has been proposed as a possible therapeutic agent against lung cancer. *Bifidobacterium infantis*-mediated sFlt-1 gene transferring system showed suppression on the tumor growth and longer survival time in C57BL/6 mice with lung cancer (Zhu et al., 2011). *Bifidobacterium infantis*-mediated soluble kinase insert domain receptor inhibited the tumor growth via increasing the necrosis rate of the tumor (Li et al., 2012). However, negative effect of microbial agents on cancer development has also been pointed out due to the potentially oncogenic toxins and metabolites by bacteria. Therefore, the combination of microbiome and its products and more conventional therapies represents novel approach for the treatment of lung cancers (Sharma et al., 2018).

### Supplementing Micronutrients to Regulate Gut Microbiota

Due to gut malabsorption, in cystic fibrosis patients, vitamin D deficiency frequently occurs (Sexauer et al., 2015). Given the vital role of vitamin D in mediating gut mucosal inflammation, vitamin D as a therapeutic approach for gut microbiota modification in cystic fibrosis has been attempted (Vanstone et al., 2015; Kanhere et al., 2018). Vitamin D can develop a healthy gut microbiota, maintain the integrity of the gut mucosal barrier, and allow beneficial bacteria to outcompete opportunistic pathogens (Kanhere et al., 2018). Enhancing intercellular junctions, reducing pro-inflammatory cytokines such as IL-8, and inhibiting apoptosis of intestinal epithelial cells might be involved in the action mechanism of vitamin D (Kanhere et al., 2018). Current evidence for the application of vitamin D in patients with cystic fibrosis is encouraging but sparse, and more sufficient evidence is expected.

Intakes of micronutrients such as vitamin C, vitamin E, niacin, beta-carotene, and riboflavin negatively correlated with the intestinal abundance of *Bacteroides* while intakes of beta-carotene and vitamin E correlated positively with Firmicutes in the setting of cystic fibrosis. Mechanism by which antioxidant vitamins influence gut microbiota still yet not being clarified (Li et al., 2017). Intakes of some flavonoids may be associated with gut microbiota variations as demonstrated by a clinical study of adult with cystic fibrosis (Li and Somerset, 2018). For example, intake of gallocatechin positively correlated with the genus Actinomyces and family Actinomycetaceae while intake of gallocatechin negatively correlated with class Coriobacteriia. Its potential impact on metabolism, immune function, and inflammation are crucial in management of cystic fibrosis patients (Li and Somerset, 2018).

Diet enriched with 5% acidic oligosaccharides led to increased bacterial clearance after *P. aeruginosa* infections in mice, resulting in limited the number and severity of pulmonary exacerbations. It stimulated the growth of bacteria involved in immunity development, such as *Sutturella wadsworthia* and *Bifidobacterium* species, and also increased the generation of butyrate and propionate (Bernard et al., 2015). Diet therapies such as probiotics and prebiotics have shown certain efficacy in improving symptom associated with chronic systemic inflammation in cystic fibrosis patients, while further study is needed to confirm this effect (Li and Somerset, 2014). Moreover the promise of other dietary strategies such as indigestible carbohydrate and modulating dietary fat to optimize nutritional status in in cystic fibrosis are also warranted (Li and Somerset, 2014).

### Cytotoxic Effect on Lung Cancer Cells

With the development of metagenomics, metatranscriptomics and culturomics platforms, increasing studies showed the promising role of probiotics in the prevention of lung cancer. The efficacies of probiotics in lung cancer cell lines, lung cancer-bearing mouse and patients with lung cancers have been studied. Two kinds of *Lactococcus lactis bacteria, L. lactis* NK34 and KC24 showed remarkable cytotoxic effect on lung cancer cells *in vitro* (Mortaz et al., 2013). In mice with non-small cell lung carcinoma, *Bacteroides fragilis* showed anti-tumor effect due to its immunostimulation of CTLA-4 blockade (Vetizou et al., 2015).

## Conclusion and Perspectives

In chronic lung diseases and respiratory infections, alternations in the composition of the intestinal and airway microbiota are presented, commonly presenting as an outgrowth of Proteobacteria and Firmicutes. A vital cross-talk between these two compartments has been noted in the setting of lung diseases. This gut–lung axis allows for the passage of endotoxins, microbial metabolites, cytokines and hormones into the bloodstream connecting the gut niche with that one of the lung. Increasing studies indicated that alterations in the gut microbial species and metabolites have been linked to changes in immune responses and inflammation as well as the disease development in the lungs. However, mechanisms by which the lung impacts the intestinal environment have not yet fully identified. By recent increasing studies, it has been demonstrated that the gut microbiota has a critical role in mediating immune responses in distant sites, including the lung. Their metabolites such as SCFAs can reach other organs via the bloodstream to exert immune regulation and anti-inflammatory effects. The direct colonization of beneficial bacteria from the gut microbiota into the airways is another possibility. As a matter of fact, due to the complex cross-talk, the causality between lung diseases and gut microbiota is still under explored. Further mechanistic insight into the pathways and mediators are expected. Nevertheless, novel therapeutic strategies that target manipulation of gut microbiome by anti-biotics, probiotics, prebiotics, natural products or diets have been tried in various lung diseases by clinical and laboratory studies. These approaches showed encouraging results in most cases. They can restore the dysbiosis of microbiota and enhance the immune responses. The effects and mechanisms of these therapeutic approaches on the overall microbiome and lung disease progression need to be understood properly by future studies.

## Author Contributions

DZ wrote the manuscript. SL revised the manuscript. NW, H-YT, and ZZ commented on the manuscript and discussed the manuscript. YF designed, revised, and finalized the manuscript.

## Conflict of Interest

The authors declare that the research was conducted in the absence of any commercial or financial relationships that could be construed as a potential conflict of interest.

## References

[B1] Abrahamsson T. R. Jakobsson H. E. Andersson A. F. Bjorksten B. Engstrand L. Jenmalm M. C. (2014). Low gut microbiota diversity in early infancy precedes asthma at school age. *Clin. Exp. Allergy* 44 842–850. 10.1111/cea.12253 24330256

[B2] Alexandre Y. Le Blay G. Boisrame-Gastrin S. Le Gall F. Hery-Arnaud G. Gouriou S. (2014). Probiotics: a new way to fight bacterial pulmonary infections? *Med. Mal. Infect.* 44 9–17. 10.1016/j.medmal.2013.05.001 23820129

[B3] Antosca K. M. Chernikova D. A. Price C. E. Ruoff K. L. Li K. Guill M. F. (2019). Altered Stool microbiota of infants with cystic fibrosis shows a reduction in genera associated with immune programming from birth. *J. Bacteriol.* 201:e00274-19. 10.1128/JB.00274-19 31209076 PMC6657602

[B4] Aragon F. Carino S. Perdigon G. de Moreno de LeBlanc A. (2015). Inhibition of growth and metastasis of breast cancer in mice by milk fermented with *Lactobacillus casei* CRL 431. *J. Immunother.* 38 185–196. 10.1097/CJI.0000000000000079 25962107

[B5] Bazett M. Bergeron M. E. Haston C. K. (2016). Streptomycin treatment alters the intestinal microbiome, pulmonary T cell profile and airway hyperresponsiveness in a cystic fibrosis mouse model. *Sci. Rep.* 6:19189. 10.1038/srep19189 26754178 PMC4709690

[B6] Begley L. Madapoosi S. Opron K. Ndum O. Baptist A. Rysso K. (2018). Gut microbiota relationships to lung function and adult asthma phenotype: a pilot study. *BMJ Open Respir. Res.* 5:e000324. 10.1136/bmjresp-2018-000324 30271607 PMC6157510

[B7] Belkaid Y. Hand T. W. (2014). Role of the microbiota in immunity and inflammation. *Cell* 157 121–141. 10.1016/j.cell.2014.03.011 24679531 PMC4056765

[B8] Berger A. K. Mainou B. A. (2018). Interactions between enteric bacteria and eukaryotic viruses impact the outcome of infection. *Viruses* 10:E19. 10.3390/v10010019 29301335 PMC5795432

[B9] Bernard H. Desseyn J. L. Bartke N. Kleinjans L. Stahl B. Belzer C. (2015). Dietary pectin-derived acidic oligosaccharides improve the pulmonary bacterial clearance of *Pseudomonas aeruginosa* lung infection in mice by modulating intestinal microbiota and immunity. *J. Infect. Dis.* 211 156–165. 10.1093/infdis/jiu391 25139019

[B10] Bruzzese E. Callegari M. L. Raia V. Viscovo S. Scotto R. Ferrari S. (2014). Disrupted intestinal microbiota and intestinal inflammation in children with cystic fibrosis and its restoration with Lactobacillus GG: a randomised clinical trial. *PLoS One* 9:e87796. 10.1371/journal.pone.0087796 24586292 PMC3929570

[B11] Budden K. F. Gellatly S. L. Wood D. L. Cooper M. A. Morrison M. Hugenholtz P. (2017). Emerging pathogenic links between microbiota and the gut-lung axis. *Nat. Rev. Microbiol.* 15 55–63. 10.1038/nrmicro.2016.142 27694885

[B12] Burke D. G. Fouhy F. Harrison M. J. Rea M. C. Cotter P. D. O’Sullivan O. (2017). The altered gut microbiota in adults with cystic fibrosis. *BMC Microbiol.* 17:58. 10.1186/s12866-017-0968-8 28279152 PMC5345154

[B13] Bush A. (2019). Pathophysiological mechanisms of asthma. *Front. Pediatr.* 7:68. 10.3389/fped.2019.00068 30941334 PMC6434661

[B14] Charlson E. S. Bittinger K. Haas A. R. Fitzgerald A. S. Frank I. Yadav A. (2011). Topographical continuity of bacterial populations in the healthy human respiratory tract. *Am. J. Respir. Crit. Care Med.* 184 957–963. 10.1164/rccm.201104-0655OC 21680950 PMC3208663

[B15] Cheng M. Qian L. Shen G. Bian G. Xu T. Xu W. (2014). Microbiota modulate tumoral immune surveillance in lung through a gammadeltaT17 immune cell-dependent mechanism. *Cancer Res.* 74 4030–4041. 10.1158/0008-5472.CAN-13-2462 24947042

[B16] Chuang S. C. Norat T. Murphy N. Olsen A. Tjonneland A. Overvad K. (2012). Fiber intake and total and cause-specific mortality in the European Prospective Investigation into Cancer and Nutrition cohort. *Am. J. Clin. Nutr.* 96 164–174. 10.3945/ajcn.111.028415 22648726

[B17] Dasari S. Kathera C. Janardhan A. Praveen Kumar A. Viswanath B. (2017). Surfacing role of probiotics in cancer prophylaxis and therapy: a systematic review. *Clin. Nutr.* 36 1465–1472. 10.1016/j.clnu.2016.11.017 27923508

[B18] de Freitas M. B. Moreira E. A. M. Tomio C. Moreno Y. M. F. Daltoe F. P. Barbosa E. (2018). Altered intestinal microbiota composition, antibiotic therapy and intestinal inflammation in children and adolescents with cystic fibrosis. *PLoS One* 13:e0198457. 10.1371/journal.pone.0198457 29933382 PMC6014676

[B19] De Lisle R. C. (2016). Decreased expression of enterocyte nutrient assimilation genes and proteins in the small intestine of cystic fibrosis mouse. *J. Pediatr. Gastroenterol. Nutr.* 62 627–634. 10.1097/MPG.0000000000001030 26551319

[B20] Debyser G. Mesuere B. Clement L. Van de Weygaert J. Van Hecke P. Duytschaever G. (2016). Faecal proteomics: a tool to investigate dysbiosis and inflammation in patients with cystic fibrosis. *J. Cyst. Fibros.* 15 242–250. 10.1016/j.jcf.2015.08.003 26330184

[B21] del Campo R. Garriga M. Perez-Aragon A. Guallarte P. Lamas A. Maiz L. (2014). Improvement of digestive health and reduction in proteobacterial populations in the gut microbiota of cystic fibrosis patients using a Lactobacillus reuteri probiotic preparation: a double blind prospective study. *J. Cyst. Fibros.* 13 716–722. 10.1016/j.jcf.2014.02.007 24636808

[B22] Demirci M. Tokman H. B. Uysal H. K. Demiryas S. Karakullukcu A. Saribas S. (2019). Reduced *Akkermansia muciniphila* and *Faecalibacterium prausnitzii* levels in the gut microbiota of children with allergic asthma. *Allergol. Immunopathol.* 47 365–371. 10.1016/j.aller.2018.12.009 30765132

[B23] Du X. Meng Q. Sharif A. Abdel-Razek O. A. Zhang L. Wang G. (2016). Surfactant proteins SP-A and SP-D ameliorate pneumonia severity and intestinal injury in a murine model of *Staphylococcus aureus* pneumonia. *Shock* 46 164–172. 10.1097/SHK.0000000000000587 26849628

[B24] Du X. Wei J. Tian D. Wu M. Yan C. Hu P. (2019). miR-182-5p contributes to intestinal injury in a murine model of *Staphylococcus aureus* pneumonia-induced sepsis via targeting surfactant protein D. *J. Cell. Physiol.* 235 563–572. 10.1002/jcp.28995 31318050

[B25] Dumas A. Bernard L. Poquet Y. Lugo-Villarino G. Neyrolles O. (2018). The role of the lung microbiota and the gut-lung axis in respiratory infectious diseases. *Cell. Microbiol.* 20:e12966. 10.1111/cmi.12966 30329198

[B26] Duytschaever G. Huys G. Bekaert M. Boulanger L. De Boeck K. Vandamme P. (2013). Dysbiosis of bifidobacteria and *Clostridium cluster* XIVa in the cystic fibrosis fecal microbiota. *J. Cyst. Fibros.* 12 206–215. 10.1016/j.jcf.2012.10.003 23151540

[B27] Elenius V. Palomares O. Waris M. Turunen R. Puhakka T. Ruckert B. (2017). The relationship of serum vitamins A, D, E and LL-37 levels with allergic status, tonsillar virus detection and immune response. *PLoS One* 12:e0172350. 10.1371/journal.pone.0172350 28235040 PMC5325266

[B28] Enaud R. Hooks K. B. Barre A. Barnetche T. Hubert C. Massot M. (2019). Intestinal inflammation in children with cystic fibrosis is associated with Crohn’s-Like microbiota disturbances. *J. Clin. Med.* 8:645. 10.3390/jcm8050645 31083321 PMC6572243

[B29] Felix K. M. Jaimez I. A. Nguyen T. V. Ma H. Raslan W. A. Klinger C. N. (2018). Gut microbiota contributes to resistance against pneumococcal pneumonia in immunodeficient Rag^(–/–)^ mice. *Front. Cell. Infect. Microbiol.* 8:118. 10.3389/fcimb.2018.00118 29755958 PMC5932343

[B30] Feng C. Feng M. Gao Y. Zhao X. Peng C. Yang X. (2018). Clinicopathologic significance of intestinal-type molecules’ expression and different EGFR gene status in pulmonary adenocarcinoma. *Appl. Immunohistochem. Mol. Morphol.* 10.1097/PAI.0000000000000632 [Epub ahead of print]. 29489510

[B31] Fontalis A. Kenanidis E. Kotronias R. A. Papachristou A. Anagnostis P. Potoupnis M. (2019). Current and emerging osteoporosis pharmacotherapy for women: state of the art therapies for preventing bone loss. *Expert Opin. Pharmacother.* 20 1123–1134. 10.1080/14656566.2019.1594772 30958709

[B32] Fouhy F. Ronan N. J. O’Sullivan O. McCarthy Y. Walsh A. Murphy M. (2017). A pilot study demonstrating the altered gut microbiota functionality in stable adults with Cystic Fibrosis. *Sci. Rep.* 7:6685. 10.1038/s41598-017-06880-y 28751714 PMC5532234

[B33] Gauguet S. D’Ortona S. Ahnger-Pier K. Duan B. Surana N. K. Lu R. (2015). Intestinal microbiota of mice influences resistance to *Staphylococcus aureus* pneumonia. *Infect. Immun.* 83 4003–4014. 10.1128/IAI.00037-15 26216419 PMC4567647

[B34] Gauvreau G. M. O’Byrne P. M. Boulet L. P. Wang Y. Cockcroft D. Bigler J. (2014). Effects of an anti-TSLP antibody on allergen-induced asthmatic responses. *N. Engl. J. Med.* 370 2102–2110. 10.1056/NEJMoa1402895 24846652

[B35] Gensollen T. Iyer S. S. Kasper D. L. Blumberg R. S. (2016). How colonization by microbiota in early life shapes the immune system. *Science* 352 539–544. 10.1126/science.aad9378 27126036 PMC5050524

[B36] Grayson M. H. Camarda L. E. Hussain S. A. Zemple S. J. Hayward M. Lam V. (2018). Intestinal microbiota disruption reduces regulatory t cells and increases respiratory viral infection mortality through increased IFNgamma PRODUction. *Front. Immunol.* 9:1587. 10.3389/fimmu.2018.01587 30042764 PMC6048222

[B37] Groves H. T. Cuthbertson L. James P. Moffatt M. F. Cox M. J. Tregoning J. S. (2018). Respiratory disease following viral lung infection alters the murine gut microbiota. *Front. Immunol.* 9:182. 10.3389/fimmu.2018.00182 29483910 PMC5816042

[B38] Gui Q. F. Lu H. F. Zhang C. X. Xu Z. R. Yang Y. H. (2015). Well-balanced commensal microbiota contributes to anti-cancer response in a lung cancer mouse model. *Genet. Mol. Res.* 14 5642–5651. 10.4238/2015.May.25.16 26125762

[B39] Haack A. Aragao G. G. Novaes M. R. (2013). Pathophysiology of cystic fibrosis and drugs used in associated digestive tract diseases. *World J. Gastroenterol.* 19 8552–8561. 10.3748/wjg.v19.i46.8552 24379572 PMC3870500

[B40] Haak B. W. Littmann E. R. Chaubard J. L. Pickard A. J. Fontana E. Adhi F. (2018). Impact of gut colonization with butyrate-producing microbiota on respiratory viral infection following allo-HCT. *Blood* 131 2978–2986. 10.1182/blood-2018-01-828996 29674425 PMC6024637

[B41] Hakozaki T. Okuma Y. Omori M. Hosomi Y. (2019). Impact of prior antibiotic use on the efficacy of nivolumab for non-small cell lung cancer. *Oncol. Lett.* 17 2946–2952. 10.3892/ol.2019.9899 30854072 PMC6365976

[B42] Hanada S. Pirzadeh M. Carver K. Y. Deng J. C. (2018). Respiratory viral infection-induced microbiome alterations and secondary bacterial pneumonia. *Front. Immunol.* 9:2640. 10.3389/fimmu.2018.02640 30505304 PMC6250824

[B43] Henriques-Normark B. Tuomanen E. I. (2013). The pneumococcus: epidemiology, microbiology, and pathogenesis. *Cold Spring Harb. Perspect. Med.* 3:a010215. 10.1101/cshperspect.a010215 23818515 PMC3685878

[B44] Hillman E. T. Lu H. Yao T. Nakatsu C. H. (2017). Microbial ecology along the gastrointestinal tract. *Microbes Environ.* 32 300–313. 10.1264/jsme2.ME17017 29129876 PMC5745014

[B45] Hu Y. Feng Y. Wu J. Liu F. Zhang Z. Hao Y. (2019a). The gut microbiome signatures discriminate healthy from pulmonary tuberculosis patients. *Front. Cell. Infect. Microbiol.* 9:90. 10.3389/fcimb.2019.00090 31001490 PMC6456665

[B46] Hu Y. Yang Q. Liu B. Dong J. Sun L. Zhu Y. (2019b). Gut microbiota associated with pulmonary tuberculosis and dysbiosis caused by anti-tuberculosis drugs. *J. Infect.* 78 317–322. 10.1016/j.jinf.2018.08.006 30107196

[B47] Huang Y. J. LiPuma J. J. (2016). The microbiome in cystic fibrosis. *Clin. Chest Med.* 37 59–67. 10.1016/j.ccm.2015.10.003 26857768 PMC5154676

[B48] Inatsu A. Kinoshita M. Nakashima H. Shimizu J. Saitoh D. Tamai S. (2009). Novel mechanism of C-reactive protein for enhancing mouse liver innate immunity. *Hepatology* 49 2044–2054. 10.1002/hep.22888 19444871

[B49] Ivashkin V. Zolnikova O. Potskherashvili N. Trukhmanov A. Kokina N. Dzhakhaya N. (2019). Metabolic activity of intestinal microflora in patients with bronchial asthma. *Clin. Pract.* 9:1126. 10.4081/cp.2019.1126 30931087 PMC6401556

[B50] Jafari S. A. Mehdizadeh-Hakkak A. Kianifar H. R. Hebrani P. Ahanchian H. Abbasnejad E. (2013). Effects of probiotics on quality of life in children with cystic fibrosis; a randomized controlled trial. *Iran. J. Pediatr.* 23 669–674. 24910746 PMC4025125

[B51] Jenne C. N. Kubes P. (2013). Immune surveillance by the liver. *Nat. Immunol.* 14 996–1006. 10.1038/ni.2691 24048121

[B52] Johnson C. C. Ownby D. R. (2017). The infant gut bacterial microbiota and risk of pediatric asthma and allergic diseases. *Transl. Res.* 179 60–70. 10.1016/j.trsl.2016.06.010 27469270 PMC5555614

[B53] Josefowicz S. Z. Niec R. E. Kim H. Y. Treuting P. Chinen T. Zheng Y. (2012). Extrathymically generated regulatory T cells control mucosal TH2 inflammation. *Nature* 482 395–399. 10.1038/nature10772 22318520 PMC3485072

[B54] Kan H. Stevens J. Heiss G. Rose K. M. London S. J. (2008). Dietary fiber, lung function, and chronic obstructive pulmonary disease in the atherosclerosis risk in communities study. *Am. J. Epidemiol.* 167 570–578. 10.1093/aje/kwm343 18063592 PMC2377022

[B55] Kanhere M. Chassaing B. Gewirtz A. T. Tangpricha V. (2018). Role of vitamin D on gut microbiota in cystic fibrosis. *J. Steroid Biochem. Mol. Biol.* 175 82–87. 10.1016/j.jsbmb.2016.11.001 27818276 PMC5415426

[B56] Keely S. Talley N. J. Hansbro P. M. (2012). Pulmonary-intestinal cross-talk in mucosal inflammatory disease. *Mucosal Immunol.* 5 7–18. 10.1038/mi.2011.55 22089028 PMC3243663

[B57] Khailova L. Baird C. H. Rush A. A. Barnes C. Wischmeyer P. E. (2017). Lactobacillus rhamnosus GG treatment improves intestinal permeability and modulates inflammatory response and homeostasis of spleen and colon in experimental model of *Pseudomonas aeruginosa* pneumonia. *Clin. Nutr.* 36 1549–1557. 10.1016/j.clnu.2016.09.025 27745813 PMC5641477

[B58] Kong Y. H. Shi Q. Han N. Zhang L. Zhang Y. Y. Gao T. X. (2016). Structural modulation of gut microbiota in rats with allergic bronchial asthma treated with recuperating lung decoction. *Biomed. Environ. Sci.* 29 574–583. 27660221 10.3967/bes2016.076

[B59] Koning C. J. Jonkers D. Smidt H. Rombouts F. Pennings H. J. Wouters E. (2010). The effect of a multispecies probiotic on the composition of the faecal microbiota and bowel habits in chronic obstructive pulmonary disease patients treated with antibiotics. *Br. J. Nutr.* 103 1452–1460. 10.1017/S0007114509993497 20021703

[B60] Kranich J. Maslowski K. M. Mackay C. R. (2011). Commensal flora and the regulation of inflammatory and autoimmune responses. *Semin. Immunol.* 23 139–145. 10.1016/j.smim.2011.01.011 21292499

[B61] Kuo S. M. (2013). The interplay between fiber and the intestinal microbiome in the inflammatory response. *Adv. Nutr.* 4 16–28. 10.3945/an.112.003046 23319119 PMC3648735

[B62] Lau A. S. Yanagisawa N. Hor Y. Y. Lew L. C. Ong J. S. Chuah L. O. (2018). *Bifidobacterium longum* BB536 alleviated upper respiratory illnesses and modulated gut microbiota profiles in Malaysian pre-school children. *Benef. Microbes* 9 61–70. 10.3920/BM2017.0063 29065707

[B63] Lee N. Kim W. U. (2017). Microbiota in T-cell homeostasis and inflammatory diseases. *Exp. Mol. Med.* 49:e340. 10.1038/emm.2017.36 28546563 PMC5454441

[B64] Li L. Krause L. Somerset S. (2017). Associations between micronutrient intakes and gut microbiota in a group of adults with cystic fibrosis. *Clin. Nutr.* 36 1097–1104. 10.1016/j.clnu.2016.06.029 27595636

[B65] Li L. Somerset S. (2014). The clinical significance of the gut microbiota in cystic fibrosis and the potential for dietary therapies. *Clin. Nutr.* 33 571–580. 10.1016/j.clnu.2014.04.004 24767984

[B66] Li L. Somerset S. (2018). Associations between flavonoid intakes and gut microbiota in a group of adults with cystic fibrosis. *Nutrients* 10:E1264. 10.3390/nu10091264 30205496 PMC6164979

[B67] Li N. Ma W. T. Pang M. Fan Q. L. Hua J. L. (2019). The commensal microbiota and viral infection: a comprehensive review. *Front. Immunol.* 10:1551. 10.3389/fimmu.2019.01551 PMC6620863 31333675

[B68] Li Z. J. Zhu H. Ma B. Y. Zhao F. Mao S. H. Liu T. G. (2012). Inhibitory effect of *Bifidobacterium infantis*-mediated sKDR prokaryotic expression system on angiogenesis and growth of Lewis lung cancer in mice. *BMC Cancer* 12:155. 10.1186/1471-2407-12-155 22536942 PMC3404897

[B69] Liu C. Yang L. Han Y. Ouyang W. Yin W. Xu F. (2019). Mast cells participate in regulation of lung-gut axis during *Staphylococcus aureus* pneumonia. *Cell Prolif.* 52:e12565. 10.1111/cpr.12565 30729611 PMC6496676

[B70] Liu T. Xiong Q. Li L. Hu Y. (2019). Intestinal microbiota predicts lung cancer patients at risk of immune-related diarrhea. *Immunotherapy* 11 385–396. 10.2217/imt-2018-0144 30693820

[B71] Luo M. Liu Y. Wu P. Luo D. X. Sun Q. Zheng H. (2017). Alternation of gut microbiota in patients with pulmonary tuberculosis. *Front. Physiol.* 8:822. 10.3389/fphys.2017.00822 29204120 PMC5698276

[B72] Luu M. Steinhoff U. Visekruna A. (2017). Functional heterogeneity of gut-resident regulatory T cells. *Clin. Transl. Immunol.* 6:e156. 10.1038/cti.2017.39 28983404 PMC5628268

[B73] Macia L. Thorburn A. N. Binge L. C. Marino E. Rogers K. E. Maslowski K. M. (2012). Microbial influences on epithelial integrity and immune function as a basis for inflammatory diseases. *Immunol. Rev.* 245 164–176. 10.1111/j.1600-065X.2011.01080.x 22168419

[B74] Maji A. Misra R. Dhakan D. B. Gupta V. Mahato N. K. Saxena R. (2018). Gut microbiome contributes to impairment of immunity in pulmonary tuberculosis patients by alteration of butyrate and propionate producers. *Environ. Microbiol.* 20 402–419. 10.1111/1462-2920.14015 29322681

[B75] Marsland B. J. Trompette A. Gollwitzer E. S. (2015). The gut-lung axis in respiratory disease. *Ann. Am. Thorac. Soc.* 12(Suppl. 2), S150–S156. 10.1513/AnnalsATS.201503-133AW 26595731

[B76] McAleer J. P. Kolls J. K. (2018). Contributions of the intestinal microbiome in lung immunity. *Eur. J. Immunol.* 48 39–49. 10.1002/eji.201646721 28776643 PMC5762407

[B77] McKeever T. M. Lewis S. A. Smith C. Hubbard R. (2002). The importance of prenatal exposures on the development of allergic disease: a birth cohort study using the West Midlands General Practice Database. *Am. J. Respir. Crit. Care Med.* 166 827–832. 10.1164/rccm.200202-158oc 12231492

[B78] Mendez R. Banerjee S. Bhattacharya S. K. Banerjee S. (2019). Lung inflammation and disease: a perspective on microbial homeostasis and metabolism. *IUBMB Life* 71 152–165. 10.1002/iub.1969 30466159 PMC6352907

[B79] Mortaz E. Adcock I. M. Folkerts G. Barnes P. J. Paul Vos A. Garssen J. (2013). Probiotics in the management of lung diseases. *Mediators Inflamm.* 2013:751068. 10.1155/2013/751068 23737654 PMC3662166

[B80] Negi S. Pahari S. Bashir H. Agrewala J. N. (2019). Gut microbiota regulates mincle mediated activation of lung dendritic cells to protect against *Mycobacterium tuberculosis*. *Front. Immunol.* 10:1142. 10.3389/fimmu.2019.01142 31231363 PMC6558411

[B81] Nielsen S. Needham B. Leach S. T. Day A. S. Jaffe A. Thomas T. (2016). Disrupted progression of the intestinal microbiota with age in children with cystic fibrosis. *Sci. Rep.* 6:24857. 10.1038/srep24857 27143104 PMC4855157

[B82] Nikniaz Z. Nikniaz L. Bilan N. Somi M. H. Faramarzi E. (2017). Does probiotic supplementation affect pulmonary exacerbation and intestinal inflammation in cystic fibrosis: a systematic review of randomized clinical trials. *World J. Pediatr.* 13 307–313. 10.1007/s12519-017-0033-6 28470579

[B83] Noverr M. C. Falkowski N. R. McDonald R. A. McKenzie A. N. Huffnagle G. B. (2005). Development of allergic airway disease in mice following antibiotic therapy and fungal microbiota increase: role of host genetics, antigen, and interleukin-13. *Infect. Immun.* 73 30–38. 10.1128/iai.73.1.30-38.2005 15618138 PMC538952

[B84] O’Dwyer D. N. Dickson R. P. Moore B. B. (2016). The lung microbiome, immunity, and the pathogenesis of chronic lung disease. *J. Immunol.* 196 4839–4847. 10.4049/jimmunol.1600279 27260767 PMC4894335

[B85] Ohnmacht C. (2016). Microbiota, regulatory T cell subsets, and allergic disorders. *Allergo J. Int.* 25 114–123. 10.1007/s40629-016-0118-0 27656354 PMC5016534

[B86] Ottiger M. Nickler M. Steuer C. Bernasconi L. Huber A. Christ-Crain M. (2018). Gut, microbiota-dependent trimethylamine-N-oxide is associated with long-term all-cause mortality in patients with exacerbated chronic obstructive pulmonary disease. *Nutrition* 45 135–141.e1. 10.1016/j.nut.2017.07.001 28870405

[B87] Park Y. Subar A. F. Hollenbeck A. Schatzkin A. (2011). Dietary fiber intake and mortality in the NIH-AARP diet and health study. *Arch. Intern. Med.* 171 1061–1068. 10.1001/archinternmed.2011.18 21321288 PMC3513325

[B88] Penders J. Stobberingh E. E. van den Brandt P. A. Thijs C. (2007). The role of the intestinal microbiota in the development of atopic disorders. *Allergy* 62 1223–1236. 10.1111/j.1398-9995.2007.01462.x 17711557

[B89] Pickard J. M. Zeng M. Y. Caruso R. Nunez G. (2017). Gut microbiota: role in pathogen colonization, immune responses, and inflammatory disease. *Immunol. Rev.* 279 70–89. 10.1111/imr.12567 28856738 PMC5657496

[B90] Qian L. J. Kang S. M. Xie J. L. Huang L. Wen Q. Fan Y. Y. (2017). Early-life gut microbial colonization shapes Th1/Th2 balance in asthma model in BALB/c mice. *BMC Microbiol.* 17:135. 10.1186/s12866-017-1044-0 28623898 PMC5473985

[B91] Rabe K. F. Watz H. (2017). Chronic obstructive pulmonary disease. *Lancet* 389 1931–1940. 10.1016/S0140-6736(17)31222-9 28513453

[B92] Rangelova E. Wefer A. Persson S. Valente R. Tanaka K. Orsini N. (2019). Surgery improves survival after neoadjuvant therapy for borderline and locally advanced pancreatic cancer: a single institution experience. *Ann. Surg.* 10.1097/SLA.0000000000003301 [Epub ahead of print]. 30946073

[B93] Ranucci G. Buccigrossi V. Borgia E. Piacentini D. Visentin F. Cantarutti L. (2018). Galacto-Oligosaccharide/Polidextrose enriched formula protects against respiratory infections in infants at high risk of atopy: a randomized clinical trial. *Nutrients* 10:E286. 10.3390/nu10030286 29494489 PMC5872704

[B94] Rogers G. B. Narkewicz M. R. Hoffman L. R. (2016). The CF gastrointestinal microbiome: structure and clinical impact. *Pediatr. Pulmonol.* 51 S35–S44. 10.1002/ppul.23544 27662102 PMC5303757

[B95] Russell S. L. Gold M. J. Hartmann M. Willing B. P. Thorson L. Wlodarska M. (2012). Early life antibiotic-driven changes in microbiota enhance susceptibility to allergic asthma. *EMBO Rep.* 13 440–447. 10.1038/embor.2012.32 22422004 PMC3343350

[B96] Saitou M. Nemoto D. Utano K. Suzuki T. Lefor A. K. Togashi K. (2018). Identification of intestinal abnormalities in patients with active pulmonary tuberculosis using small bowel capsule endoscopy. *Endosc. Int. Open* 6 E1103–E1108. 10.1055/a-0655-2086 30211298 PMC6133679

[B97] Samuelson D. R. Charles T. P. de la Rua N. M. Taylor C. M. Blanchard E. E. Luo M. (2016). Analysis of the intestinal microbial community and inferred functional capacities during the host response to *Pneumocystis pneumonia*. *Exp. Lung Res.* 42 425–439. 10.1080/01902148.2016.1258442 27925857 PMC5304582

[B98] Scales B. S. Dickson R. P. Huffnagle G. B. (2016). A tale of two sites: how inflammation can reshape the microbiomes of the gut and lungs. *J. Leukoc. Biol.* 100 943–950. 10.1189/jlb.3mr0316-106r 27365534 PMC5069096

[B99] Schippa S. Iebba V. Santangelo F. Gagliardi A. De Biase R. V. Stamato A. (2013). Cystic fibrosis transmembrane conductance regulator (CFTR) allelic variants relate to shifts in faecal microbiota of cystic fibrosis patients. *PLoS One* 8:e61176. 10.1371/journal.pone.0061176 23613805 PMC3629184

[B100] Schuijt T. J. Lankelma J. M. Scicluna B. P. de Sousa e Melo F. Roelofs J. J. de Boer J. D. (2016). The gut microbiota plays a protective role in the host defence against pneumococcal pneumonia. *Gut* 65 575–583. 10.1136/gutjnl-2015-309728 26511795 PMC4819612

[B101] Sexauer W. P. Hadeh A. Ohman-Strickland P. A. Zanni R. L. Varlotta L. Holsclaw D. (2015). Vitamin D deficiency is associated with pulmonary dysfunction in cystic fibrosis. *J. Cyst. Fibros.* 14 497–506. 10.1016/j.jcf.2014.12.006 25577127

[B102] Sharma A. Viswanath B. Park Y. S. (2018). Role of probiotics in the management of lung cancer and related diseases: an update. *J. Funct. Foods* 40 625–633. 10.1016/j.jff.2017.11.050

[B103] Stiemsma L. T. Turvey S. E. (2017). Asthma and the microbiome: defining the critical window in early life. *Allergy Asthma Clin. Immunol.* 13:3. 10.1186/s13223-016-0173-6 28077947 PMC5217603

[B104] Stokholm J. Blaser M. J. Thorsen J. Rasmussen M. A. Waage J. Vinding R. K. (2018). Maturation of the gut microbiome and risk of asthma in childhood. *Nat. Commun.* 9:141. 10.1038/s41467-017-02573-2 29321519 PMC5762761

[B105] Tanasienko O. A. Cheremshenko N. L. Titova G. P. Potebnya M. G. Gavrilenko M. M. Nagorna S. S. (2005). Elevation of the efficacy of antitumor vaccine prepared on the base of lectines from *B. subtilis* B-7025 upon its combined application with probiotics in vivo. *Exp. Oncol.* 27 336–338. 16404358

[B106] Tarashi S. Ahmadi Badi S. Moshiri A. Nasehi M. Fateh A. Vaziri F. (2018). The human microbiota in pulmonary tuberculosis: not so innocent bystanders. *Tuberculosis* 113 215–221. 10.1016/j.tube.2018.10.010 30514505

[B107] Troseid M. Ueland T. Hov J. R. Svardal A. Gregersen I. Dahl C. P. (2015). Microbiota-dependent metabolite trimethylamine-N-oxide is associated with disease severity and survival of patients with chronic heart failure. *J. Intern. Med.* 277 717–726. 10.1111/joim.12328 25382824

[B108] Tsang M. S. Cheng S. W. Zhu J. Atli K. Chan B. C. Liu D. (2018). Anti-inflammatory activities of pentaherbs formula and its influence on gut microbiota in allergic asthma. *Molecules* 23:E2776. 10.3390/molecules23112776 30373169 PMC6278535

[B109] Tweedle J. L. Deepe G. S. Jr. (2018). Tumor necrosis factor alpha antagonism reveals a gut/lung axis that amplifies regulatory T cells in a pulmonary fungal infection. *Infect. Immun.* 86:e00109-18. 10.1128/IAI.00109-18 29581197 PMC5964519

[B110] Vanstone M. B. Egan M. E. Zhang J. H. Carpenter T. O. (2015). Association between serum 25-hydroxyvitamin D level and pulmonary exacerbations in cystic fibrosis. *Pediatr. Pulmonol.* 50 441–446. 10.1002/ppul.23161 25657016

[B111] Varraso R. Willett W. C. Camargo C. A. Jr. (2010). Prospective study of dietary fiber and risk of chronic obstructive pulmonary disease among US women and men. *Am. J. Epidemiol.* 171 776–784. 10.1093/aje/kwp455 20172921 PMC2877480

[B112] Vernocchi P. Del Chierico F. Quagliariello A. Ercolini D. Lucidi V. Putignani L. (2017). A metagenomic and in silico functional prediction of gut microbiota profiles may concur in discovering new cystic fibrosis patient-targeted probiotics. *Nutrients* 9:1342. 10.3390/nu9121342 29232848 PMC5748792

[B113] Vernocchi P. Del Chierico F. Russo A. Majo F. Rossitto M. Valerio M. (2018). Gut microbiota signatures in cystic fibrosis: loss of host CFTR function drives the microbiota enterophenotype. *PLoS One* 13:e0208171. 10.1371/journal.pone.0208171 30521551 PMC6283533

[B114] Vetizou M. Pitt J. M. Daillere R. Lepage P. Waldschmitt N. Flament C. (2015). Anticancer immunotherapy by CTLA-4 blockade relies on the gut microbiota. *Science* 350 1079–1084. 10.1126/science.aad1329 26541610 PMC4721659

[B115] Vieira A. T. Rocha V. M. Tavares L. Garcia C. C. Teixeira M. M. Oliveira S. C. (2016). Control of *Klebsiella pneumoniae* pulmonary infection and immunomodulation by oral treatment with the commensal probiotic *Bifidobacterium longum* 5(1A). *Microbes Infect.* 18 180–189. 10.1016/j.micinf.2015.10.008 26548605

[B116] Wang H. Lian P. Niu X. Zhao L. Mu X. Feng B. (2018). TLR4 deficiency reduces pulmonary resistance to *Streptococcus pneumoniae* in gut microbiota-disrupted mice. *PLoS One* 13:e0209183. 10.1371/journal.pone.0209183 30562386 PMC6298678

[B117] Xin X. Dai W. Wu J. Fang L. Zhao M. Zhang P. (2016). Mechanism of intestinal mucosal barrier dysfunction in a rat model of chronic obstructive pulmonary disease: an observational study. *Exp. Ther. Med.* 12 1331–1336. 10.3892/etm.2016.3493 27588054 PMC4997986

[B118] Young R. P. Hopkins R. J. Marsland B. (2016). The gut-liver-lung axis. Modulation of the innate immune response and its possible role in chronic obstructive pulmonary disease. *Am. J. Respir. Cell Mol. Biol.* 54 161–169. 10.1165/rcmb.2015-0250PS 26473323

[B119] Zhang H. Yeh C. Jin Z. Ding L. Liu B. Y. Zhang L. (2018). Prospective study of probiotic supplementation results in immune stimulation and improvement of upper respiratory infection rate. *Synth. Syst. Biotechnol.* 3 113–120. 10.1016/j.synbio.2018.03.001 29900424 PMC5995450

[B120] Zhang W. Q. Zhao S. K. Luo J. W. Dong X. P. Hao Y. T. Li H. (2018). Alterations of fecal bacterial communities in patients with lung cancer. *Am. J. Transl. Res.* 10 3171–3185. 30416659 PMC6220220

[B121] Zhang Y. Li T. Yuan H. Pan W. Dai Q. (2018). Correlations of inflammatory factors with intestinal flora and gastrointestinal incommensurate symptoms in children with asthma. *Med. Sci. Monit.* 24 7975–7979. 10.12659/MSM.910854 30401793 PMC6234755

[B122] Zhu H. Li Z. Mao S. Ma B. Zhou S. Deng L. (2011). Antitumor effect of sFlt-1 gene therapy system mediated by *Bifidobacterium infantis* on lewis lung cancer in mice. *Cancer Gene Ther.* 18 884–896. 10.1038/cgt.2011.57 21921942 PMC3215997

[B123] Zhuang H. Cheng L. Wang Y. Zhang Y. K. Zhao M. F. Liang G. D. (2019). Dysbiosis of the gut microbiome in lung cancer. *Front. Cell. Infect. Microbiol.* 9:112. 10.3389/fcimb.2019.00112 31065547 PMC6489541

[B124] Zitvogel L. Daillere R. Roberti M. P. Routy B. Kroemer G. (2017). Anticancer effects of the microbiome and its products. *Nat. Rev. Microbiol.* 15 465–478. 10.1038/nrmicro.2017.44 28529325

